# Supplementary material for: Unconstrained multivariate EEG decoding can help detect lexical-semantic processing in individual children
Source: Sci Rep. 2020 Jul 2;10:10849. doi: 10.1038/s41598-020-67407-6 (PMC7331680; doi:10.1038/s41598-020-67407-6)
Supplement: Supplementary file 1 — Supplementary file1 (PDF 252 kb) [file 41598_2020_67407_MOESM1_ESM.pdf]

Unconstrained multivariate EEG decoding can help detect lexical-semantic processing in individual children

Authors, affiliations

Selene Petit[*,a,b], Nicholas A. Badcock[a,b], Tijl Grootswagers[a,b,c] , and Alexandra Woolgar[a,b,d]

a. Department of Cognitive Science, Macquarie University, Australia

b. ARC Centre of Excellence in Cognition and its Disorders (CCD)

c. School of Psychology, University of Sydney, Australia

d. Medical Research Council (UK), Cognition and Brain Sciences Unit, University of Cambridge, Cambridge, UK

*. Corresponding author at: Department of Cognitive Science, Macquarie University, Australia. Selene.petit@mq.edu.au.

1. Supplementary Table 1: List of stimuli

Sentence frame	Congruent target	Incongruent target
While eating Steve accidentally bit his	tongue	kite
It was windy enough to fly a	kite	tongue
The directions did not match any roads on the old	map	pace
Jessie ran the race at a slower	pace	map
At the royal wedding the princess married the	prince	chair
The man sat down on the comfortable	chair	prince
For her school dance Becky wore a new	dress	bridge
The boat passed easily under the	bridge	dress
The little girl was very afraid of the	dark	bread
She went to the bakery to buy a loaf of	bread	dark
The dentist says to brush your teeth twice a	day	band
She played the guitar so she joined the	band	day
The student went to the library to read a	book	paint
He wanted color in the room so he bought a tin of	paint	book
The squirrel stored nuts in the	tree	bike
A good way to exercise is to ride a	bike	tree
During the lecture Jen kept checking the	time	queen
The princess may someday become a	queen	time
After dinner they washed the	dishes	trees
In autumn leaves fall off the	trees	dishes
Most students prefer to work during the	day	tea
I put the kettle on to make a hot cup of	tea	day
After pulling him over, the police officer told the man to get out of his	car	book
He turned the page of his favorite	book	car
She forgot her watch so she asked for the	time	book
He read a chapter of the	book	time
She got out of the car and closed the	door	cake
There were candles on the birthday	cake	door
I prefer cats rather than	dogs	teeth
The dentist opened her mouth to check her	teeth	dogs
For his wife's birthday he baked a	cake	time
Her job was easy most of the	time	cake
At night the old woman locked the	door	break
Several hours into his shift Lyle was ready for a	break	door
During the winter holidays people tend to eat a lot of good	food	door
I had no key to open the	door	food
His father was the coach of the football	team	door
After coming inside Bob locked the	door	team
The indoor plant was growing bigger and needed a new	pot	broom
John swept the floor with a	broom	pot
The moon was shining in the night	sky	vase
Katie put the flowers in a big	vase	sky
She put on her sunglasses to protect her tired	eyes	marks

Due to his hard work he received excellent	marks	eyes
The surfer is scared of being bitten by a great white	shark	ink
The cheap pen quickly ran out of black	ink	shark
The dragon was slain by the valiant	knight	stick
To help her walk around the girl used a walking	stick	knight
Father carved the turkey with a sharp	knife	ink
The pen in his pocket had unfortunately leaked	ink	knife
He washed his hands with water and	soap	knife
At dinner he cut his steak with a sharp	knife	soap
He had a long day and was in a bad	mood	splash
She jumped in the pool and made a big	splash	mood
To keep the dogs out of the yard he built a six foot	fence	snack
Before practice he decided to eat a quick	snack	fence
She made him a sandwich for his packed	lunch	cell
The prisoner fell asleep inside his dark	cell	lunch
She looked up at night to see a sky full of bright	stars	aim
Tom’s arrows missed the target due to his bad	aim	stars
For breakfast he ate scrambled	eggs	socks
Derek’s feet were cold so he put on some thick	socks	eggs
Cid needed a belt to hold up his	pants	teeth
After every meal it’s good to brush your	teeth	pants
His shirt was so worn it had a big	hole	walk
The old man has to use a cane to go on a short	walk	hole
The genie granted the man his first	wish	sky
The stars are high up in the night	sky	wish
There were no extra seats so she sat on the concrete	floor	milk
The little girl left Santa a plate of cookies and	milk	floor
She could tell he was mad by the tone of his loud	voice	milk
The hungry baby wanted to drink	milk	voice
He went to the lake to catch a big	fish	wall
She hung the painting up on the white	wall	fish
He took his dog out for a short	walk	sun
The baseball player’s cap protected him from the hot	sun	walk
I roasted the marshmallow over the hot	fire	ring
Bob proposed and gave her a diamond	ring	fire
It was dark in the room so she turned on the bright	light	ring
The man presented his new fiancée with an expensive diamond	ring	light
Sarah saw animals from around the world at the country's best	zoo	sword
The knight got ready for battle and drew his magnificent	sword	zoo
To cut the chicken Sue needed a sharp	knife	farm
They raised pigs on their private	farm	knife
Tim joined the airforce because he always wanted to fly a	plane	desk
To do her homework she sat down at her	desk	plane
The real estate agent sold the big	house	word
Today the baby spoke his first	word	house
Most cats see very well at	night	floor

I accidentally spilled some water on the concrete	floor	night
After driving for hours Kevin rested at the next	stop	hand
When he touched the hot stove the child burned his right	hand	stop
Pete broke his arm and needed to wear a	cast	pan
Jane fried some bacon in the	pan	cast
